# Combined esophageal injury complicated by progression to a second perforation: a case report

**DOI:** 10.4076/1752-1947-3-9213

**Published:** 2009-09-11

**Authors:** Andreas Krieg, Christoph Vogt, Uwe Ramp, Ludger W Poll, Martin J Brinkmann, Edwin Bölke, Wolfram T Knoefel, Matthias Peiper

**Affiliations:** 1 Department of General, Visceral and Pediatric Surgery, University Hospital Düsseldorf, Heinrich-Heine-University Düsseldorf, D-40225 Düsseldorf, Germany; 2 Department of Gastroenterology, Hepatology and Infectiology, University Hospital Düsseldorf, Heinrich-Heine-University Düsseldorf, D-40225 Düsseldorf, Germany; 3 Institute of Pathology, University Hospital Düsseldorf, Heinrich-Heine-University Düsseldorf, D-40225 Düsseldorf, Germany; 4 Institute of Diagnostic Radiology, University Hospital Düsseldorf, Heinrich-Heine-University Düsseldorf, D-40225 Düsseldorf, Germany; 5 Department of Radiation Oncology, University Hospital Düsseldorf, Heinrich-Heine-University Düsseldorf, D-40225 Düsseldorf, Germany

## Abstract

**Introduction:**

Intramural dissection of the esophagus is a rare disorder characterized by a lesion between the submucosa and mucosa dividing the esophagus into a false and true lumen. The etiology of esophageal dissection remains uncertain but it affects predominantly women in their seventies and eighties. Symptoms may include uncharacteristic ones such as retrosternal pain, odynophagia or dysphagia. Conservative management is thought to be adequate and surgery should only be performed if complications such as abscess formation or perforation appear. Here we report the case and surgical management of a combined esophageal perforation and dissection.

**Case presentation:**

We report the case of a combined esophageal perforation and dissection in a 45-year-old Caucasian woman with a history of relapsing periods of dysphagia since her childhood. The clinical course in this patient was complicated by progression to a second perforation, which made a definitive surgical management by esophagectomy necessary.

**Conclusion:**

To the best of our knowledge, this is the first reported case of a combined esophageal perforation and dissection complicated by progression to a second perforation. This emphasizes that cautious and intensive observation is necessary in patients with esophageal dissection.

## Introduction

According to their extent, esophageal injuries are classified into (i) transmural, short and predominantly distally localized perforations, (ii) mucosal, short lesions in the distal esophagus, and (iii) intramural dissections [1]-[3]. Intramural esophageal dissection is a rare disorder characterized by the appearance of a false lumen between the esophageal mucosa and submucosa separated by a mucosal septum. Predominantly, the dissection occurs in women in their seventies and eighties [1]. Symptoms such as sudden retrosternal pain, hematemesis and odynophagia have been described [4]. The pathogenesis is as yet unknown but it has been postulated that submucosal bleeding, which secondarily perforates the mucosa and by this decompresses the intramural hematoma or a primarily existing mucosal tear with secondary submucosal dissection might be an explanation for the development of a transmural dissection [4]. Diagnostic procedures involve an esophagogram with contrast, endoscopy or computed tomography (CT) [5].

Here we report a very rare case of transmural esophageal dissection with complete transmural perforation after endoscopic recovery of an impacted pearl onion (typically less than 25 mm in diameter and also known as silver or cocktail onions) in a patient suffering from chronic dysphagia since her childhood. To the best of our knowledge, this is the first reported case of esophageal dissection progressing to complete perforation.

## Case presentation

**Figure 1:**
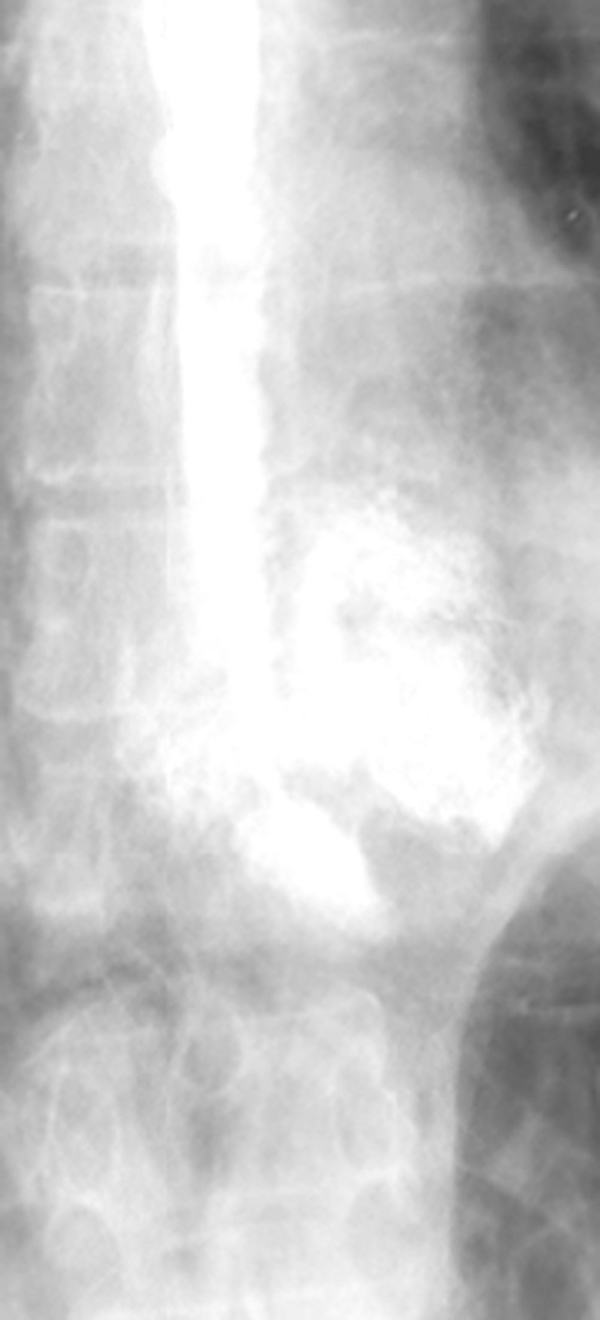
**An esophagogram showing a distal perforation with contrast extravasation**.

A 45-year-old German Caucasian woman was transferred to our department of general surgery with a suspected esophageal perforation after endoscopic recovery of a pearl onion which was impacted in the middle third of the esophagus. After recovery of the pearl onion, upper gastrointestinal endoscopy revealed slightly bleeding mucosa at the site of impaction as well as an impassable stenosis. Her medical history, included relapsing periods of dysphagia since her childhood that were never examined by endoscopy or gastrografin swallow. At admission, she presented in a stable condition with normal laboratory findings. Clinical investigation revealed no abnormalities other than retrosternal pain as well as emphysema of the skin. Chest and abdominal radiography showed free intra-abdominal air as well as a pneumomediastinum. A gastrografin swallow was performed revealing leakage at the distal esophagus and pneumoperitoneum (Figure 1). Explorative laparotomy and direct closure with hemifundoplication were performed and the mediastinum was drained.

**Figure 2:**
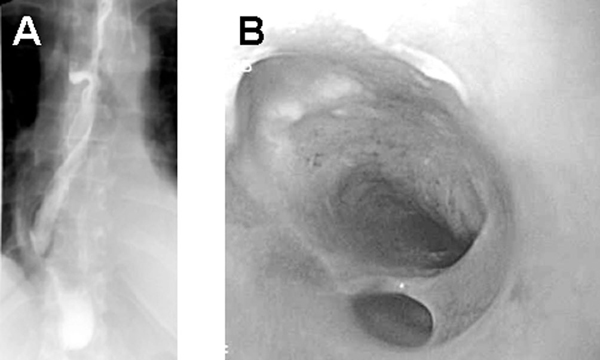
****(A)** An esophagogram indicating mucosal irregularities as well as the double-barreled appearance of the esophageal lumen by intramural dissection**. **(B)** Upper gastrointestinal endoscopy revealed esophageal dissection by identifying a small true lumen separated from an expanded false lumen with multiple ulcerations.

Because of pathological drainage continuing on the tenth postoperative day as well as increasing white cell counts, we performed a gastrografin swallow that revealed mucosal irregularities and a double-barreled esophagus (Figure 2A). Upper gastrointestinal endoscopy identified an esophageal intramural dissection with complete obstruction of the true lumen at 35 cm, as well as mucosal bridges (Figure 2B).

These findings left us without any feasible conservative management options. The presence of mediastinitis due to a suspected transmural perforation prompted us to perform a transhiatal esophagectomy with cervical esophagostoma and blind closure of the stomach.

**Figure 3:**
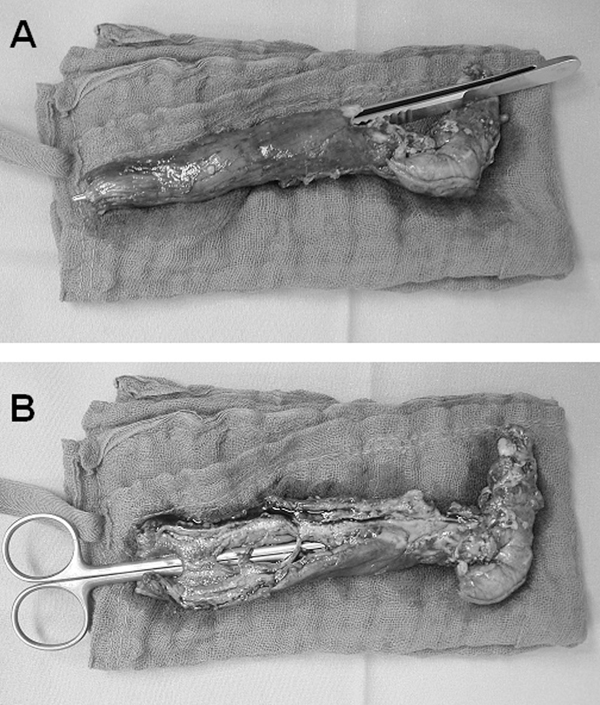
**Esophageal specimen after discontinuing resection**. **(A)** To label the distal perforation, forceps are brought into the distal transmural perforation. **(B)** Scissors are inlaid into the true lumen and stretch the mucosal bridges.

The pathology report showed the true esophageal lumen and a transmural perforation as well as a second lumen that was focally covered by a flat squamous epithelium with multiple ulcerations within the submucosal layer (Figure 3). The postoperative course was uneventful and 3 months after esophagectomy, a reconstruction with a cervical esophagogastric anastomosis was performed and the patient was discharged on the 12th postoperative day after an uneventful course.

## Discussion

The etiology of esophageal dissection still remains unclear. Two pathogenetic theories have been postulated [4]. The first proposes that intramural dissection occurs from bleeding in the submucosa which secondarily tears the mucosa. The second theory favors that the mucosa tears first with secondary dissection of the submucosa. Nevertheless, extensive esophageal intramural formation of hematoma has been reported in patients with caverno-capillary hemangiomatosis in the lamina propria or disorders in blood coagulation. Vomiting might also lead to esophageal dissection. An abnormal swallowing mechanism may be another cause, but in some patients, the cause of this lesion remains unclear. Although the pathology report and gastrografin swallow revealed no esophageal stenosis or abnormal swallowing mechanism, our patient reported a history of recurrent dysphagia during food ingestion since her early childhood.

Because symptoms such as severe central chest pain might be uncharacteristic, more frequent diseases such as myocardial infarction, aortic dissection, or gastrointestinal ulcer are often initially suspected [4].

Diagnostic principles in esophageal intramural dissection include radiological contrast swallow, CT of the chest and/or endoscopy of the upper gastrointestinal tract [5,6]. Typical signs of esophageal dissection in contrast swallows are a so-called double-barreled esophagus due to contrast filling of both the true and false lumen separated by a mucosal bridge which appears as a thin lucent line, or the mucosal strip sign. The use of water-soluble contrast media such as gastrografin is preferred because, as observed in our patient, a simultaneous perforation might be present. However, in some cases, especially if submucosal formation of a hematoma is present, gastrografin might not be able to enter the false lumen. CT can demonstrate the intramural extent of a hematoma, esophageal wall thickening and air collections, and might distinguish between the false and true lumen by visualization of a mucosal septum. Upper gastrointestinal endoscopy is not only a safe technique in the diagnosis of esophageal dissection but might also be useful as a therapeutic approach [7].

Intramural dissection of the esophagus has a good prognosis and can be managed conservatively with initial intravenous fluid and nutrition [8]. If, during the course, no fever is present, laboratory findings reveal a decrease in inflammatory parameters and dysphagia or odynophagia are improving, oral intake can be started with fluids. Endoscopic incision of mucosal bridges with a diathermy has been described if the mucosal wall persists and the patient's symptoms of dysphagia do not disappear [7]. If complications such as perforation, septic mediastinitis or abscess formation occur, surgical therapy such as esophagectomy or drainage might become necessary, as shown in this patient.

In our patient, it is speculative whether esophageal dissection originated from abnormal swallowing after impaction of a pearl onion or due to the endoscopic procedure. We hypothesize that the initial trauma permitted esophageal dissection as well as distal perforation. The false lumen probably remained initially invisible because of intramural formation of a hematoma that prevented contrast media from entering it. This theory is supported by the histological examination of the resected esophagus which identified a partial epithelial lining in the false lumen and which may occur after several days.

## Conclusion

To the best of our knowledge, we have described the first case of a combined esophageal dissection and perforation complicated by progression to a second perforation which underlines that cautious and intensive observation is necessary in patients with esophageal dissection.

## Abbreviation

CT: computed tomography.

## Consent

Written informed consent was obtained from the patient for publication of this case report and any accompanying images. A copy of the written consent is available for review by the Editor-in-Chief of this journal.

## Competing interests

The authors declare that they have no competing interests.

## Authors' contributions

All authors analyzed and interpreted the patient data. UR performed the histological examination of the organs. AK, EB and MP were major contributors in writing the manuscript. All authors read and approved the final manuscript.
